# Symptom patterns in the daily life of PSC patients

**DOI:** 10.1111/liv.15271

**Published:** 2022-04-18

**Authors:** Kim N. van Munster, Marcel G. W. Dijkgraaf, Ronald P. J. Oude Elferink, Ulrich Beuers, Cyriel Y. Ponsioen

**Affiliations:** ^1^ Department of Gastroenterology and Hepatology, Amsterdam UMC University of Amsterdam Amsterdam The Netherlands; ^2^ Clinical Research Unit, Amsterdam UMC University of Amsterdam Amsterdam The Netherlands; ^3^ Tytgat Laboratory for Liver and Intestinal Diseases Amsterdam University Medical Centers University of Amsterdam Amsterdam the Netherlands

**Keywords:** cholestatic symptoms, diurnal pattern, experience sampling method, high‐density mapping, mobile app

## Abstract

**Background & Aims:**

Patients with primary sclerosing cholangitis (PSC) may suffer from complaints such as pruritus, right upper abdominal quadrant pain (RUQ‐A) and fatigue. However, the severity of these complaints, daily and/or seasonal patterns and other factors of influence in PSC are largely unknown. The aim of this study is to assess daily symptoms and patterns thereof in PSC patients in their natural setting.

**Methods:**

A mobile application was designed according to the experience sampling method. Push notifications with a response time of max 4 h were sent during tiers of 3 months. Questions comprised VAS scales on degree of pruritus, fatigue, RUQ‐A, time of the day these symptoms were worst, as well as time of intake of medication. Linear mixed modelling was used to identify patient‐ and external factors associated with pruritus, fatigue and RUQ‐A pain.

**Results:**

A total of 6713 questionnaires were completed by 137 patients. Fatigue was the most prevalent symptom among PSC patients being reported in a striking 71% of measurements, followed by pruritus (38%). Both increased during the day and were associated with longer disease duration. A highly significant correlation between pruritus and day temperature was observed (ρ = −0.14, *p* = .000), and itch was generally worse during winter (*p* = .000). Patient preference for the tool was high.

**Conclusion:**

Pruritus and fatigue are prevalent symptoms in the daily life of PSC patients and show a distinct diurnal pattern. This may have implications for efficient dosing of anti‐pruritic agents. The level of pruritus is highly correlated with day temperature, which may have several implications.

**Abbreviations**
AIC: Akaike's information criterion
AIH: autoimmune hepatitis
ATX: autotaxin
EASL: European Association for the Study of the Liver
ESM: experience sampling method
IBD: inflammatory bowel disease
KNMI: Koninklijk Nederlands Meteorologisch Instituut
LD‐PSC: large duct primary sclerosing cholangitis
LPA: lysophosphatidic acid
LTx: liver transplantation
PROM: patient‐reported outcome measure
PSC: primary sclerosing cholangitis
RUQ‐A: right upper quadrant abdominal
SD: standard deviation
TRP: transient receptor potential
UDCA: ursodeoxycholic acid
UV: ultraviolet
VAS: visual analogue scale

**Lay summary**

Patients with primary sclerosing cholangitis frequently suffer from complaints such as itching, pain in the right upper quadrant of their belly, fatigue and sometimes bouts of fever. However, the severity of these complaints and daily and seasonal patterns affecting individuals are unknown. In this study, we measured these complaints frequently by using a mobile app with push messages and evaluated the influence of external factors such as weather condition. Fatigue was present in more than 70% of patients, followed by itching in 40%. Itch was usually most severe in the evening and correlated with lower temperature, especially during winter. The mobile app proved highly satisfactory to users.

## 1 INTRODUCTION

Primary sclerosing cholangitis (PSC) is a chronic disease characterized by inflammation and progressive destruction of bile ducts. It is an orphan disease with unknown aetiology, although the commonly co‐existing inflammatory bowel disease (IBD) suggests that it is an immune dysbalance disease with a pathogenetic mechanism originating in the colon. There is no curative treatment available except for liver transplantation.

Natural course of PSC is highly variable and unpredictable, but in general, patients report significant impact on both their social and working life.^1^ Presence and intensity of symptoms are important drivers of health‐related quality of life and disease burden in PSC.^1^ Moreover, in some liver transplant programmes, untreatable pruritus can in exceptional situations be an indication for transplantation.^2^

Impact of PSC on quality of life has been studied to some extent, but very little research has been done regarding prevalence and severity of symptoms. In a Swedish population‐based study from 1996, 56% of patients were symptomatic at diagnosis.^3^ More recently, only 28% of patients were symptomatic at diagnosis in a Finnish tertiary cohort.^4^ Pruritus, fatigue and right upper quadrant abdominal (RUQ‐A) pain are the most prevalent symptoms.

Prevalence of pruritus ranges from 22% to 36% in different studies and patient surveys.^4^, ^5^, ^6^ Most patients report a peak incidence of pruritus in the evening and early night. A seasonal pattern with an increase in wintertime was suggested in one study.^7^ Pathophysiology of pruritus in (cholestatic) liver disease was increasingly studied over the past decades leading to several new therapeutic agents.^8^ Bile salts,^9^ lysophosphatidic acid (LPA) and autotaxin (ATX),^10^ endogenous opoids^11^ and bilirubin^12^ may play a role, though none of them seem to be the dominant mechanism. Patients with therapy refractory cholestatic pruritus are experimentally treated with ultraviolet(UV)‐B phototherapy suggesting a possible role of (sun)light in pruritus pathophysiology.^13^

Data on prevalence and severity of fatigue are conflicting; some studies report fatigue in 40% to 66% of PSC patients,^4^ while in the study of Bjornsson et al.,^14^ fatigue was not playing an important role in PSC patients and fatigue severity scores were similar to (or even better than) the general population. Pathophysiology of fatigue has been investigated in PBC, pointing to a central and a peripheral component resulting in both cognitive impairment and physical weakness.^15^

Insight in the prevalence and severity of symptoms, day‐ and seasonal patterns and other factors of influence is important to properly inform patients and to unravel underlying pathophysiological mechanisms. So far, studies evaluating symptoms in PSC had highly variable populations, that is, prevalence of liver cirrhosis ranged from 5% to 62%. Moreover, all studies were based on one single measurement during hospital visits, and external factors such as daytime, season and medication intake were not taken into account. Hence, there is a clear need for longitudinal prospective data with high‐density mapping of daily symptoms in the home setting.

The experience sampling method (ESM) is a study design frequently used in psychiatry to high‐density monitoring of subjective reactions and experiences in the patients' natural environment.^16^ Short patient‐reported outcome questionnaires are sent to electronic devices, such as mobile phones, at pre‐selected randomized time‐points.^17^ ESM has been proven to be a feasible and valid method to evaluate subjective outcomes more reliably than retrospective data collection.^18^

The aim of this prospective study is to assess daily symptoms in PSC patients in a high‐density mapping fashion to evaluate patterns and factors of influence using ESM.

## 2 METHODS

### 2.1 Study design

Data were collected prospectively and intermittently between February 2018 and August 2020 using the experience sampling method. All patients from the Dutch population‐based EpiPSC2 cohort were approached for participation. Inclusion criteria were a confirmed PSC diagnosis (according to EASL clinical practice guidelines) and age ≥18 years.^19^ PSC patients that underwent liver transplantation were included in the study, but questionnaires completed after LT were analysed separately. Patients without a smartphone with access to Apple App Store or Android Play Store could not participate.

### 2.2 Data collection

The ESM is a sampling method based on repeated issuing of patient‐reported outcome questionnaires in the natural environment setting. For this study, a specific mobile application (PSC app) was designed. Patients received a push notification if a new questionnaire was available. To avoid that, patients would fill in most questionnaires by the end of the day, questionnaires were only available for 4 h. Data were collected during a period of 3 months followed by a few months without questionnaires to keep compliance high. Patients could participate in one single period or in more (maximum 4) sampling periods. A predefined data collection schedule was made based on 5 different questionnaires in 4 different timeslots. Questionnaires sent at 8 AM were available until noon, questionnaires sent at noon were available until 4 PM, questionnaires sent at 4 PM were available until 8 PM and questionnaires sent at 8 PM were available until midnight. Answers were directly stored in a cloud‐based data collection platform CastorEDC. The PSC app and content management system MoniQ were both built by EverywhereIM. Both EverywhereIM and CastorEDC adhere to NEN7510, ISO9001 and 27 001:2013.

A Visual Analogue Scale (VAS) – presented as slider from 0 to 10 – was used to measure severity of complaints at that moment. “*How severe is your itch at this moment?*” for pruritus, *“How severe is your fatigue at this moment?”* for fatigue and *“How severe is your pain in the right upper quadrant of the abdomen at this moment?”* for RUQ‐A pain. In case of no pruritus, fatigue or RUQ‐A pain, patients could set the slider to 0 or check the box “not applicable”. Five different questionnaires were sent to patients. The exact date and time of completing a questionnaire was automatically stored.

The following questionnaires were sent:

1. *All complaints*, with a VAS for pruritus, fatigue and RUQ‐A pain.
2. *Pruritus, fatigue or RUQ‐A pain during the day*, with a VAS and the question “At what time in the past 24 h the itch/fatigue/RUQ‐A pain was worst?” and “How severe was it at that moment?”. This type of question was used to accommodate for night‐time complaints and to get an impression on patient's subjective appraisal of his/her peak moment of complaints.
3. *Pruritus and medication intake*, with a VAS for pruritus and the question “If applicable, at what time did you take your last ursodeoxycholic acid and/or your last anti‐pruritic medication?”

Data coming from different questionnaires were combined to make optimal use of the available data. All measurements after liver transplantation were excluded from the aggregated analyses and only used for the subgroup analyses *complaints after liver transplantation*. Patients who never reported a VAS >1 of a specific symptom were considered never to have it. Patients who always reported a VAS >2 of a specific symptom were considered always to have it. Daytime was categorized in morning (8:00 AM–11:59 AM), afternoon (noon–5:59 PM) and evening (6:00 PM–midnight).

Clinical data were accrued from medical chart reviews. Patients with IBD‐Unspecified (IBD‐U) were handled as UC patients in the analysis as there were very little IBD‐U patients. Data on weather conditions were retrieved from the Royal Dutch Meteorological Institute (KNMI). Because the Netherlands is a small and densely populated country, these data were considered representative for the cohort studied.

### 2.3 Statistical analysis

Pearson's Chi‐square test was used for categorical data. Spearman's rank correlation was used for correlations between not normally distributed data. T‐test or Mann–Whitney U test was used for continuous data, depending on the distribution of data. If data were not normally distributed but median (range) would not be informative to report, data were presented as mean (SD), but non‐parametric tests were used for the analyses. A *p*‐value of <0.05 was considered statistically significant.

Demographics were presented for responders (at least one questionnaire completed) and non‐responders (never responded). Attrition analysis was performed to evaluate responder bias.

Linear mixed models were used to identify patient‐ and external factors associated with severity of pruritus, fatigue and RUQ‐A pain. Natural cubic splines were used to model time. Covariates were first analysed in univariate analyses, and multivariate analysis was performed using backward selection based on Akaike's information criterion (AIC). Estimates and 95% confidence intervals were reported, as well as P‐values.

An additional analysis evaluating the effect of the same patient‐ and external factors on the presence of pruritus, fatigue and RUQ‐A pain was performed as sensitivity analysis. A VAS of 0 or 1 was scored as “no pruritus, fatigue or RUQ‐A pain respectively” and a VAS >1 was scored as “presence of pruritus, fatigue or RUQ‐A pain respectively”.

## 3 RESULTS

### 3.1 Baseline characteristics

**FIGURE 1:**
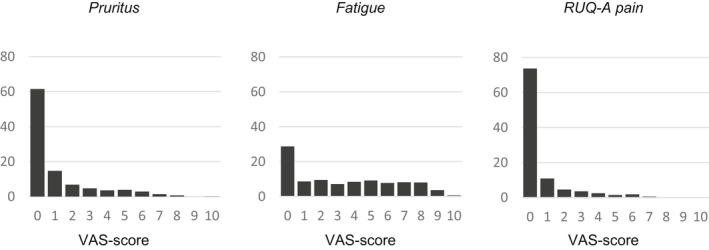
VAS score distribution of pruritus, fatigue and RUQ‐A pain

A total of 162 patients was included in the study, of which 137 (85%) responded at least once. Baseline characteristics of both responders and non‐responders are presented in Table 1. Median number completed questionnaires per patients was 50 (120). Mean follow‐up period was 35 weeks. Sampling characteristics are presented in Table S1. A total of 15 874 questionnaires were sent, of which 6713 (42%) were completed. Visual analogue score distribution of pruritus, fatigue and RUQ‐A pain is presented in Figure 1. Pruritus score distribution was based on 2444 measurements in 120 different patients. It was absent in 62% of measurements and was rarely higher than 7 (<1%). Fatigue score distribution was based on 1652 measurements in 120 patients. In 29% of measurements, no fatigue was reported, and in 20%, VAS was above 7. RUQ‐A score distribution was based on 1591 measurements in 120 patients. It was absent in 74% of measurements and higher than 7 in <1%. The correlation between degree of pruritus and fatigue was 0.40 (0.34;0.46) (Table 2). Correlation between pruritus and RUQ‐A pain was 0.43 (0.36;0.49) and correlation between fatigue and RUQ‐A pain was 0.43 (0.38, 0.49). These correlations are classified as moderate.

**TABLE 1:** Demographics

	Responders *n* = 137	Non‐responders *n* = 25	*p*‐value
	*n* (%)	*n* (%)	
Male gender	89 (65)	16 (64)	.926
Age at start^a^	51 *±* 13	51 *±* 16	.819
PSC type			.605
LD‐PSC	114 (83)	22 (88)	
PSC‐AIH	9 (7)	2 (8)	
SD‐PSC	14 (10)	1 (4)	
Disease duration, years^2^	11 ±37)	13 ±37)	.651
ALP >1.3x ULN at diagnosis	36 (59)	4 (40)	.261
Co‐existing IBD	88 (65)	16 (64)	.946
IBD type			.809
Ulcerative colitis	61 (69)	12 (75)	
Crohn's disease	22 (25)	4 (25)	
IBD‐U	5 (6)	0 (0)	
P‐HBI / p‐SCCAI at start^2^	1 12)	1.5 ±6	.883
At baseline			
Colectomy	13 (10)	1 (4)	.369
HPB malignancy	5 (4)	0 (0)	.332
Liver transplant	17 (12)	5 (20)	.308
End‐stage liver disease	17 (12)	3 (12)	.954
During study period			
Colectomy	1 (1)		
HPB malignancy	1 (1)		
Liver transplant	3 (2)		
End‐stage liver disease	18 (13)		
UDCA use	121 (88)		
Anti‐pruritus medication	19 (14)		
Fibrates	7 (5)		
Rifampicin	8 (6)		
Naltrexon	6 (4)		
Sertralin	2 (2)		

^a^ Mean (±SD) ^2^ Median (range).

**TABLE 2:** Factors associated with severity of pruritus, fatigue and RUQ‐A pain

	Univariate		Final model	
	Est.	95% CI	Est.	95% CI
**Pruritus**				
Female gender	−1.3	(−7.1; 4.5)		
Age	0.1	(−0.1; 0.3)		
PSC type (LD‐PSC = ref) PSC‐AIH	−4.0	(−14.9; 6.9)		
SD‐PSC	2.8	(−6.3; 11.9)		
IBD type (No‐IBD = ref) UC	3.8	(−2.5; 10.1)		
CD	0.9	(−7.2; 8.9)		
Disease duration, years	0.3	(−0.1; 0.6)		
Clinical signs dec. cirrhosis	3.8	(−0.9; 16.6)		
Daypart (morning = ref) Afternoon	−1.0	(−2.1; 0.2)	−0.8	(−2.0; 0.3)
Evening	0.5	(−0.8; 1.8)	0.6	(−0.6; 1.9)
Day temperature	−0.2*	(−0.3; ‐0.1)	−0.2*	(−0.3; −0.0)
Sun hours per day	−0.1	(−0.2; 0.1)		
Humidity	6.7	(−0.1; 0.1)		

	Est.	95% CI	Est.	95% CI
**Fatigue**				
Female gender	13.2*	(3.7; 22.6)	14.8*	(5.7; 23.8)
Age	−0.1	(−0.5; 0.3)		
PSC type (LD‐PSC = ref) PSC‐AIH	20.6*	(2.0; 39.2)	19.8*	(2.5; 37.2)
SD‐PSC	8.7	(−5.7; 23.0)	12.6	(−0.9; 26.1)
IBD type (No‐IBD = ref) UC	2.5	(−8.0; 13.1)		
CD	−0.4	(−14.1; 13.3)		
Disease duration, years	0.5	(−0.1; 1.0)	0.6*	(0.1; 1.1)
Clinical signs dec. cirrhosis	6.8	(−16.7; 30.3)		
Daypart (morning = ref) Afternoon	4.5*	(2.6; 6.3)	4.5*	(2.7; 6.3)
Evening	7.7*	(5.7; 9.6)	7.6*	(5.6; 9.6)
Day temperature	0.03	(−0.2; 0.2)		
Cloud coverage	0.7*	(0.4; 1.1)		

	Est.	95% CI	Est.	95% CI
**RUQ‐A pain**				
Female gender	3.3	(−1.1; 7.7)	3.1	(−1.2; 7.4)
Age	0.1	(−0.1; 0.3)		
PSC type (LD‐PSC = ref) PSC‐AIH	5.2	(−3.4; 13.8		
SD‐PSC	1.2	(−5.6; 8.0)		
IBD type (No‐IBD = ref) UC	−2.8	(−7.6; 2.0)	−1.8	(−6.5; 2.8)
CD	−7.0*	(−13.2; ‐0.8)	−5.8	(−11.8; 0.3)
Disease duration, years	−0.04	(−0.3; 0.2)		
Clinical signs dec. cirrhosis	−0.8	(−10.4; 8.8)		
Daypart (morning = ref) Afternoon	−0.2	(−1.4; 1.1)		
Evening	0.1	(−1.2; 1.4)		
Day temperature	−0.1	(−0.2; 0.0))		
Cloud coverage	0.0	(−0.2; 0.3)		

* *p* < .05.

The presence of pruritus was never reported by 47 patients (40%) and always by 10 patients (8%). Eighteen patients (15%) never reported fatigue, while 46 (39%) always reported fatigue. RUQ‐A pain was reported by 74 patients (62%) and always by 3 patients (3%).Degree of pruritus increased significantly depending on disease duration (ρ = 0.9, *p* = .000) (Figure S1). Severity of fatigue also increased significantly (ρ = 0.12, *p* = .000). No correlation between RUQ‐A pain and disease duration was observed (ρ = −0.01, *p* = .935).

A total of 1026 questionnaires were completed by 20 individual patients after liver transplantation. One patient had signs of recurrent PSC and was excluded from the analyses. The remaining 19 patients completed a total of 991 questionnaires. Half of those patients had very limited symptoms (mean VAS pruritus, fatigue and RUQ‐A pain<2). Fatigue was always present in 16% of patients and never in 21%. Pruritus and RUQ‐A pain was never present in 58% and 95% of patients, respectively. Two patients had persistent high scores on pruritus and RUQ‐A pain; these patients had substantial co‐morbidities (i.e. kidney failure, diabetes, IBD, Bechterew).

### 3.2 Day pattern of symptoms

**FIGURE 2:**
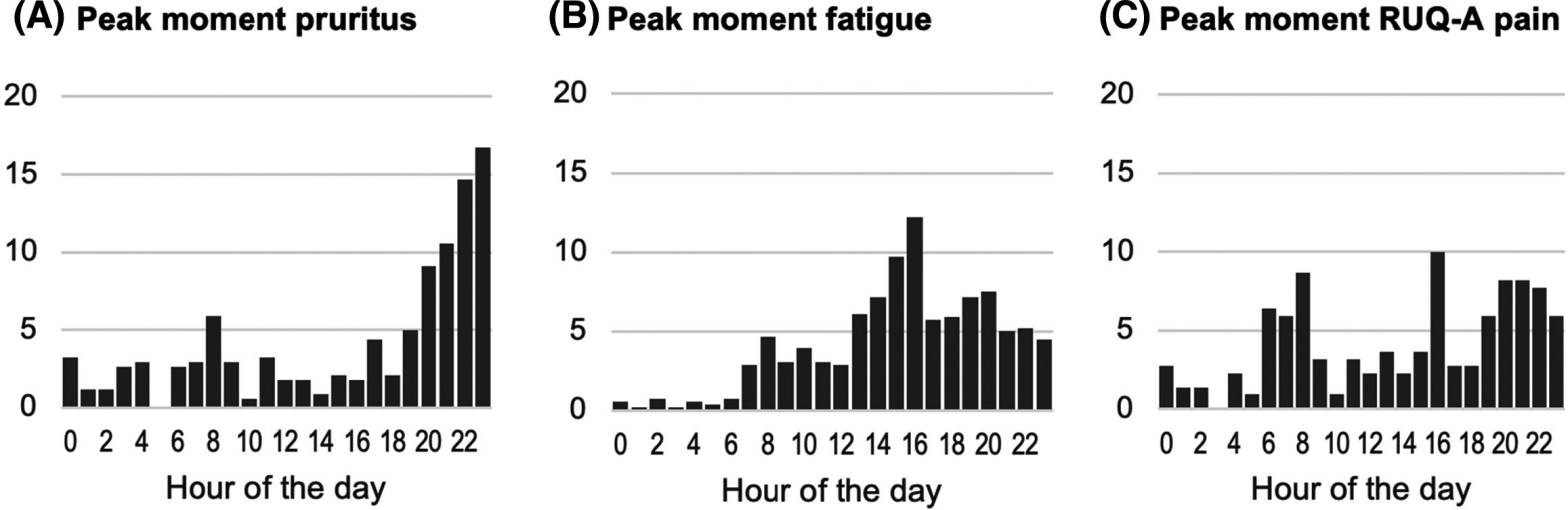
Patients' reported moment of most severe pruritus (A), fatigue (B) and RUQ‐A pain (C) in the previous 24 h

Peak moment of pruritus, fatigue and RUQ‐A in the previous 24 h as reported by patients are presented in Figure 2a–c. In 56%, pruritus was reported to be worst in the evening, after 7 PM. A second peak was observed at 8 AM in the morning.

Fatigue was reported to be worst in afternoon, around 4 PM. RUQ‐A pain was reported to be worst in the morning (6–8 AM) and evening (7–11 PM).

Day of the week had no effect on severity of pruritus, fatigue and RUQ‐A pain. Severity of symptoms was also similar on working days (Monday to Friday) as compared to weekend days (Saturday and Sunday).

### 3.3 Pruritus and medication intake

Mean pruritus score per hour since previous UDCA intake is presented in Figure S2. No correlation between time since previous UDCA intake and degree of pruritus was found (ρ = −0.003, *p* = .941).

### 3.4 Seasonal variation and impact of weather conditions on symptoms

**FIGURE 3:**
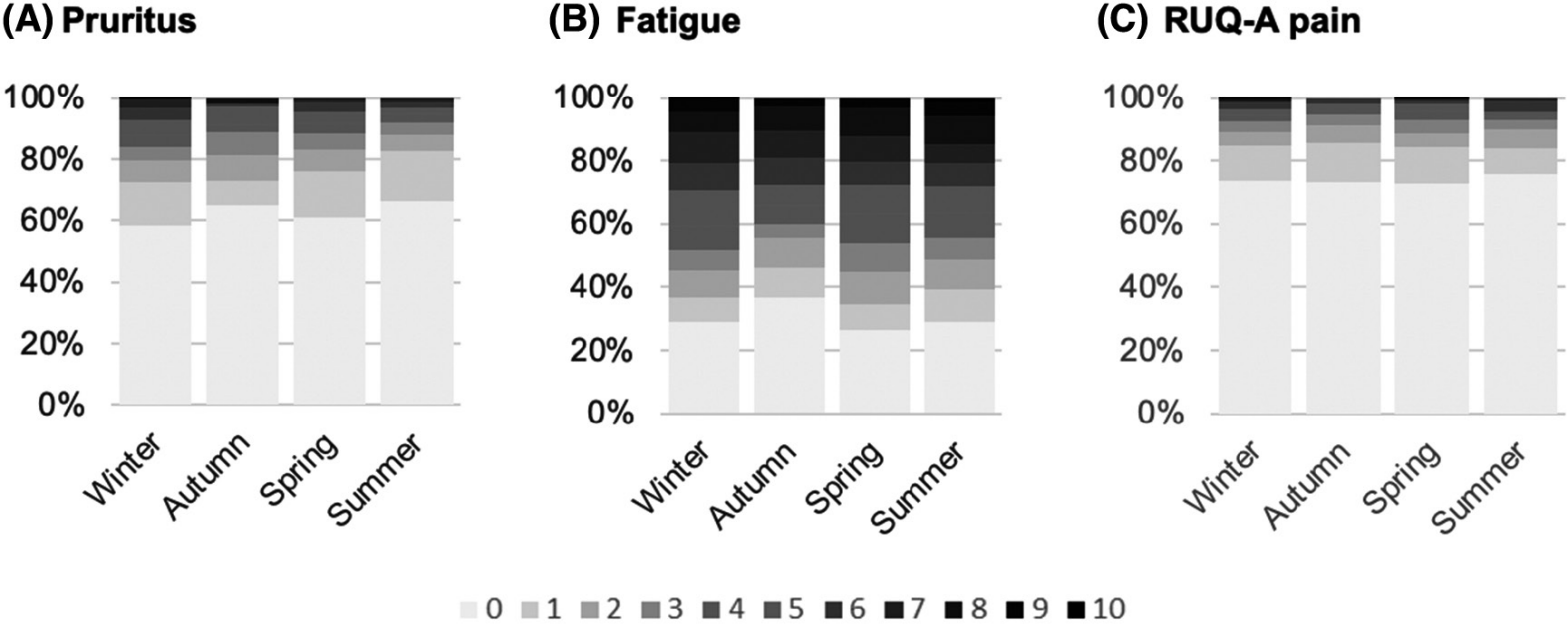
Severity of pruritus (A), fatigue (B) and RUQ‐A pain (C) in winter, autumn, spring and summer measured by VAS

A significant association between season and severity of pruritus was observed with pruritus more severe during the winter (*p* = .000,Figure 3). Mean (SD) pruritus score was 1.3 (2.1), 1.0 (1.8), 1.0 (1.8) and 0.9 (1.6) in winter, autumn, spring and summer, respectively. No significant association between season and fatigue or RUQ‐A pain was observed.

**FIGURE 4:**
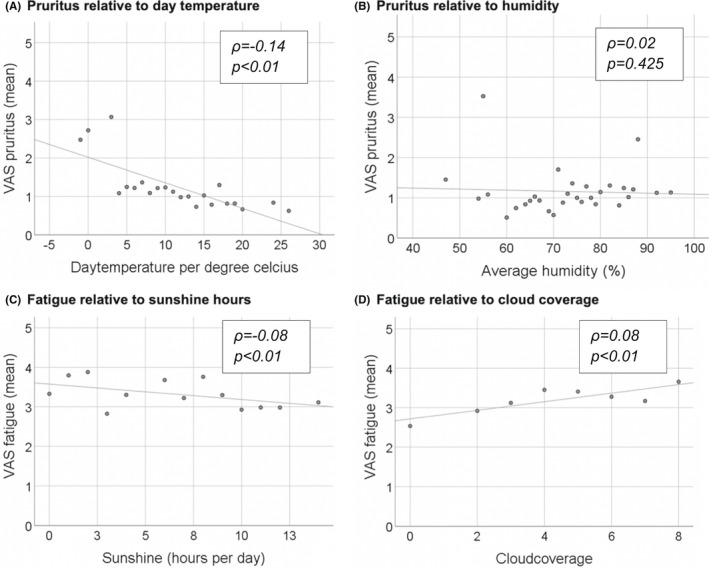
Association between pruritus and day temperature (A) and humidity (B) and fatigue and sunshine hours (C) and cloud coverage (D)

A negative correlation was observed between mean day temperature and degree of pruritus, ρ = −0.14, *p* = .000 (Figure 4,Table S2). No significant correlation between humidity and pruritus was observed, but mean pruritus score was highest at very low humidity (55%) and very high humidity (90%). Severity of fatigue correlated with number of sunshine hours per day (ρ = −0.08, *p* = .005), humidity (ρ = 0.08, *p* = .002) and degree of cloud coverage (ρ = 0.08, *p* = .005). No significant correlation between any of the weather conditions and RUQ‐A pain was observed.

### 3.5 Multivariate analyses

In the multivariate linear mixed model analysis, no patient‐related factors were associated with pruritus (Table 2). However, mean day temperature correlated negatively with pruritus (Est. = −0.2[−0.3;‐0.0] *p* = .005).

Patient‐related factors associated with increased fatigue were female gender (Est. = 14.8 [5.7;23.8] *p* = .002), PSC‐AIH (relative to LD‐PSC) (Est. = 19.8[2.5;37.2] *p* = .026) and disease duration (Est. = 0.6[0.1,1.1] *p* = .024) (Table 2).

Female patients reported higher RUQ‐A pain scores compared to male patients (Est. = 3.1[−1.2;7.4] *p* = .155) (Table 2). Patients with Crohn's disease (relative to no IBD) reported lower RUQ‐A scores (Est. = −5.8[−11.8;0.3] *p* = .062).

A sensitivity analysis evaluating the effect of the same factors on presence (instead of severity) of pruritus, fatigue and RUQ‐A pain (VAS 0–1 vs. VAS ≥2) yielded similar results (data not shown).

### 3.6 Patient preference

Most patients (66%) completed a single questionnaire in less than 30 s; only 8% of patients spent more than 1 min (Figure S3). The three‐month data collection period was good for most patients (86%) and too short for the other 14%. Only 6% of patients sometimes experienced technical issues, 1% had often technical issues. None of the participants preferred questionnaires on paper or by email above the smartphone app. In particular, 63% rated data collection by a smartphone app as 'much better'.

## 4 DISCUSSION

We here present the first ever experience sampling study in PSC patients evaluating daily symptoms. The results show that cholestatic symptoms are quite common. Fatigue was the most prevalent symptom among PSC patients being reported in a striking 71% of measurements, followed by pruritus (38%). Degree of symptoms correlated with each other, indicating that patients often suffer from more than one symptom at the time. Level of symptoms increases as disease duration becomes longer, which seems logical but has not been quantified before. Fatigue was worse in the afternoon and evening, which is understandable. With regard to diurnal pattern of pruritus, both the multivariate analysis and respondent's own perception of worst level of pruritus showed that this was most severe during the evening.

No correlation between time since previous UDCA intake and degree of pruritus was observed, which is in line with the study of *van de Meeberg* et al.,^20^ in which UDCA saturation of bile was similar in patients with a single dose compared to patients with multiple doses per day.

No patient‐related factors were associated with pruritus. Patients with PSC‐AIH, female gender, and longer disease duration reported significantly more fatigue. Interestingly, both fatigue and pruritus were not dependent of IBD status, and RUQ‐A pain was even lower in patients with Crohn's disease (relative to no IBD). Although we encouraged patients to report any form of abdominal pain in the right upper quadrant, we cannot exclude that the latter might be a result of CD patients not reporting RUQ‐A as they related it to IBD and not PSC. After liver transplantation, fatigue was the most prevalent symptom, present in 50% of individuals. More than half of post‐LTx patients never reported pruritus and almost none had RUQ‐A pain after LTx.

Interestingly, a highly significant correlation between pruritus and day temperature was observed. Mean pruritus score decreased when temperature increased. One other study described an increase in cholestatic pruritus during wintertime.^7^ Since it is well established that dry skin leads to or aggravates pruritus, this effect could possibly be attributed to dry air because of heating, but the effect of (outside) day temperature is irrespective of (outside) humidity.^21^ Moreover, mean pruritus score decreases gradually when temperature increased, also above 15 degrees (when internal heating will be limited). The most dramatic decrease of perceived pruritus with increasing outside temperature was found between 0 and 12 °C, a range at which it may be expected that patients wear warmer clothing. Hence, under these conditions, the skin temperature may actually be higher than the skin temperature with summer clothing. This will be particularly the case when patients are indoors where the ambient temperature is kept relatively constant and humidity is lower. Assuming that this is the case, this observation fits with the current ideas about the mechanism of itch signal transduction. Itch is signalled through small diameter C‐fibres that are very similar to (and possibly overlap with) those C‐fibres that transmit inflammatory pain. Initiation of a full action potential in these neurons appears to depend on primary or secondary activation of TRP channels like TRPA1, TRPV1 and TRPV4.^22^, ^23^, ^24^ In mice with a deleted TRPV1 or TRPA1 channel gene, several forms of itch are reduced.^25^ The influx of cations causes a depolarization that elicits a full action potential. Important in this context is that several TRP channels including TRPA1 and TRPV1 and TRV4 are also temperature‐sensitive receptors that are activated at their specific temperature ranges (TRPA1 is activated at increasing temperature around 10°C, TRPV4 around 30°C and TRPV1 around 40°C). Hence, increased body temperature will enhance an itch signal.

The impact of inside and outside temperature, inside and outside humidity and moment of the day on severity of pruritus and fatigue should be taken into account in future trials evaluating symptom modifying drugs.

Patients' organizations stress the importance of research regarding symptoms and quality of life in PSC. A survey among PSC patients in the UK indicated that 78% of patients with symptoms were very interested in taking part in clinical trials investigating symptomatic therapies. Recent data from our group among 328 respondents revealed that health‐related quality of life of PSC patients can be substantially impaired by pruritus (manuscript submitted). Therefore, measuring the degree of symptoms longitudinally and in patients' natural setting is important for the management of patients and for clinical trials on disease‐ and symptom‐modifying drugs.

This form of data collection by a mobile application was highly valued by patients; almost all patients preferred it above questionnaires by email or on paper. Future studies measuring patient‐reported outcome measures should consider using this feasible and valid data collection method.

The experience sampling method has many benefits. Data are collected in the patients' natural environment which results in higher ecological validity of data.^26^ Patients are asked about their current complaints precluding recall bias.^27^ The effect of sampling error is greatly reduced by the multiple repeated measurements, and the enormous amount of data gives the opportunity to clarify patterns over time. Nevertheless, there are some limitations that need to be addressed. Owing to the questionnaires' limited availability of 4 h (to ensure questionnaires were completed equally throughout the day), the rate of missing data is relatively high (57%). However, baseline characteristics of responders were comparable to those of non‐responders.

Although VAS was validated for pruritus, fatigue and pain, use of these questionnaires in the ESM setting with a mobile application was not validated. In a previous study, we have issued the same PROM via a digital survey and the mobile application within a few days to evaluate reproducibility, which yielded highly correlating results. Intraclass correlation coefficient ranged from 0.69 to 0.92 in 69 patients.^28^ Lastly, some patients reported that they had the idea that they got more itchy because of frequent questions about it.

In conclusion, this first ever experience sampling method by a mobile application in PSC patients showed that pruritus and fatigue are quite prevalent symptoms in this disease and show a distinct daily pattern with increase in the evening. This may have implications for efficient dosing of anti‐pruritic agents. However, time since previous UDCA administration did not correlate with severity of itch. The level of pruritus highly correlated with day temperature, which might be a clue for the unravelling of the pathophysiological mechanism of pruritus. Furthermore, this method of accruing data on symptoms and medication intake is highly appreciated by patients, feasible and reproducible.

## ETHICS APPROVAL

This study was approved nationally by the IRB of the University Medical Centre Utrecht, NL14614.041.06. All participating patients gave written informed consent. The study is part of the EpiPSC2 project registered in the Dutch Trial Registry under NTR 2813.

## CONFLICT OF INTEREST

CYP has received research grant funding from Takeda, Gilead and Perspectum, advisory fees from Pliant and Shire and speaker's fees from Takeda, Tillotts. KNM, MGD, ROE and UB declare no conflicts of interest.

## Supporting information


Figure S1
