# Supplementary material for: Symptom patterns in the daily life of PSC patients
Source: Liver Int. 2022 Apr 18;42(7):1562–70. doi: 10.1111/liv.15271 (PMC9325051; doi:10.1111/liv.15271)
Supplement: Supplementary file 1 — Figure S1 [file LIV-42-1562-s001.docx]

Supplementary tables and figures

**Supplementary table 1**. Sampling characteristics

|  | Sent *n* | Completed *n (%)* |
| --- | --- | --- |
| Questionnaire |  |  |
| Pruritus | 6706 | 2886 (43%) |
| Fatigue | 4671 | 1959 (42%) |
| RUQ-A pain | 4497 | 1868 (42%) |
| Moment of the day |  |  |
| Morning | 3390 | 1575 (39%) |
| Afternoon | 5988 | 3121 (52%) |
| Evening | 5896 | 2017 (34%) |
| Season |  |  |
| Winter | 4651 | 2394 (51%) |
| Autumn | 1764 | 370 (21%) |
| Spring | 5720 | 2303 (40%) |
| Summer | 3739 | 1646 (44%) |
|  |  |  |

**
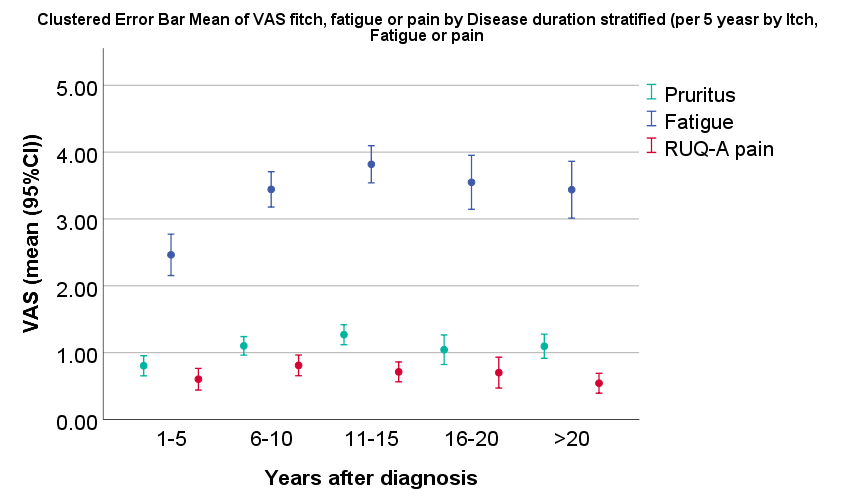
**

**Supplementary figure 1.** Mean VAS(95%CI) of pruritus, fatigue and RUQ-pain grouped by disease duration after diagnosis

| 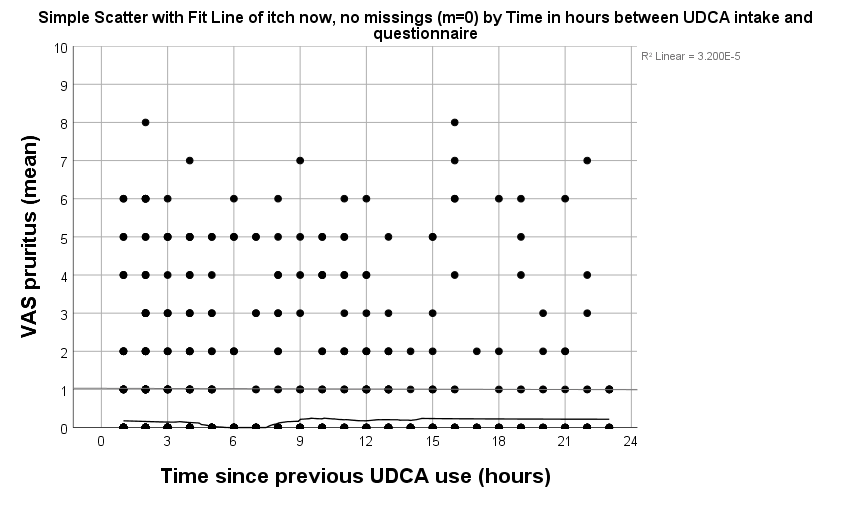 |
| --- |

**Supplementary figure 2.** Pruritus relative to time since previous UDCA use

**Supplementary table 2.** Correlations between symptoms and weather conditions

|  | ***Pruritus*** | | ***Fatigue*** | | ***RUQ-A pain*** | |
| --- | --- | --- | --- | --- | --- | --- |
|  | r | p-value | r | p-value | r | p-value |
| Temperature | -0.14 | 0.000 | -0.02 | NS | 0.00 | NS |
| Humidity | 0.03 | NS | 0.08 | 0.002 | -0.01 | NS |
| Cloud coverage | -0.00 | NS | 0.08 | 0.005 | 0.02 | NS |
| Precipitation | -0.02 | NS | 0.04 | NS | -0.03 | NS |
| Sun hours | -0.02 | NS | -0.08 | 0.005 | -0.02 | NS |

| **a. Time to fill in one questionnaire** | **b. Data collection period of 3 months** |
| --- | --- |
|  |  |
| **c. Technical issues** | **d. Data collection by mobile app**  **(instead of email or paper)** |
|  |  |

**Supplementary figure 3.** Patients' experience PSC App
